# Ideas, concerns, expectations, and effects on life (ICEE) in GP consultations: an observational study using video-recorded UK consultations

**DOI:** 10.3399/BJGPO.2023.0008

**Published:** 2023-09-06

**Authors:** Peter J Edwards, Grace M Sellers, Isabel Leach, Lydia Holt, Matthew J Ridd, Rupert A Payne, Rebecca K Barnes

**Affiliations:** 1 Centre for Academic Primary Care, Bristol Medical School, University of Bristol, Bristol, UK; 2 Royal Devon University Healthcare NHS Foundation Trust, Exeter, UK; 3 University of Sheffield Medical School, University of Sheffield, Sheffield, UK; 4 National Institute for Health and Care Research Health Protection Research Unit, University of Bristol, Bristol, UK; 5 Exeter Collaboration for Academic Primary Care, University of Exeter Medical School, University of Exeter, Exeter, UK; 6 Nuffield Department of Primary Care Health Sciences, University of Oxford, Oxford, UK

**Keywords:** consultation skills, family medicine, patient perspectives, patient-centered care, general practice, primary health care

## Abstract

**Background:**

Eliciting patients’ ideas, concerns, expectations, and whether a problem has an 'effect' on their life (ICEE), is a widely recommended communication technique. However, it is not known how frequently ICEE components are raised in UK GP consultations.

**Aim:**

To assess the frequency of ICEE in routine GP consultations with adult patients and explore variables associated with ICEE.

**Design & setting:**

An observational study was undertaken. It involved secondary analysis of a pre-existing archive of video-recorded, face-to-face GP consultations in the UK.

**Method:**

Observational coding of 92 consultations took place. Associations were assessed using binomial and ordered logistic regression.

**Results:**

Most consultations included at least one ICEE component (90.2%). The most common ICEE component per consultation was patient ideas (79.3%), followed by concerns (55.4%), expectations (51.1%), and then effects on life (42.4%). For all ICEE components patients more commonly initiated the ICEE dialogue, and in only three consultations (3.3%) did GPs directly ask patients about their expectations. Problems that were acute (odds ratio [OR] 2.98, 95% confidence interval [CI] = 1.36 to 6.53, *P* = 0.007) or assessed by GPs aged ≥50 years (OR 2.10, 95% CI = 1.07 to 4.13, *P* = 0.030) were associated with more ICEE components. Problems assessed later in the consultation (OR 0.60 per problem order increase, 95% CI = 0.41 to 0.87, *P* = 0.007) by patients aged ≥75 years (OR 0.40, 95% CI = 0.16 to 0.98, *P* = 0.046) and from the most deprived cohort (OR 0.39, 95% CI = 0.17 to 0.92, *P* = 0.032) were associated with fewer ICEE components. Patient ideas were associated with more patients being ‘very satisfied’ post-consultation (OR 10.74, 95% CI = 1.60 to 72.0, *P* = 0.014) and the opposite was true of concerns (OR 0.14, 95% CI = 0.02 to 0.86, *P* = 0.034).

**Conclusion:**

ICEE components were associated with patient satisfaction and demographic variables. Further research is required to assess if the way ICEE are communicated affects these associations and other potential confounders.

## How this fits in

Eliciting patients' ICEE is widely recommended in patient-centred consulting. However, it is not known how often ICEE occurs in UK GP consultations, and the international evidence on whether this affects patient outcomes is mixed. In this study, nine in ten consultations with adult patients presenting with at least one new problem included ≥1 ICEE components, although it was far more common for the patient to raise an ICEE component themselves rather than the GP directly asking about them. There were more ICEE components mentioned for problems that were acute and assessed by older GPs, and there were fewer ICEE components for problems assessed later in the consultation, by patients aged ≥75 years and living in the most deprived neighbourhoods, compared with the least deprived. The voicing of patient ideas was associated with increased patient satisfaction scores, whereas the opposite was true of patient concerns.

## Introduction

Contemporary evidence-based medicine involves using the best available evidence to create tailored management plans that consider individual patient preferences and values.^1^ Byrne and Long’s seminal work in 1976^2^ was one of the earliest studies to highlight the importance of discovering the patient’s agenda for the consultation.^3^ Subsequently, multiple consultation models, including Pendleton *et al*’s,^4^ Neighbour’s,^5^ and the Calgary–Cambridge model,^6^ have described a structured approach to understanding the patient perspective within healthcare encounters. Common to all these models is exploring patients’ ideas, concerns, and expectations (ICE). How the condition has ‘effects’ on the patient’s day-to-day life is sometimes considered as an additional element of patient-centred consulting (ICEE).^7^

Initial reports suggested patient-centred consulting was associated with increased patient satisfaction.^8^ However, a 2012 Cochrane review concluded that interventions teaching clinicians patient-centred communication skills had mixed effects on satisfaction and health behaviour.^9^ One difficulty uncovered in this review was knowing which component(s) of patient-centred care were having an effect. Additionally, patients and doctors have voiced dissatisfaction with the tick-box use of ICE.^10,11^ This is consistent with a 2021 study conducted in Germany that found no correlation between patient-centred communication and patient satisfaction.^12^

Previous observational studies have reported on the utility of ICE in primary care consultations from Canada^13^ and Belgium.^14^ While exploring ICEE is considered best practice in UK GP training,^15^ its frequency in practice remains unknown. This study aimed to develop a standardised framework for reporting and classifying talk about ICEE during healthcare encounters. Using this framework, the authors aimed to determine the frequency of ICEE in video-recorded, face-to-face GP consultations, compare verbal interactions with medical records, explore factors associated with ICEE, and explore its relationship with patient satisfaction. These findings could then inform clinical practice and future research, including identifying populations less likely to communicate ICEE and how closely medical records reflect what has happened in the consultation.

## Method

### Study design

This was an observational study involving secondary analysis of a pre-existing archive of video-recorded, face-to-face UK GP consultations, with linked electronic health records (EHRs) and survey data.

### Data

Data were obtained from the One in a Million primary care consultations archive.^16^ Full details of the archive are reported elsewhere.^17^ In brief, of 421 adult patients (aged ≥18 years) invited to participate, 327 recordings of unselected routine consultations with 23 different GPs working across 12 West of England practices were obtained between 2014 and 2015. A total of 318 patients gave consent for their consultation data to be reused by the original research team. All consultations that included at least one ‘new’ (first presentation) problem were included (*n* = 97). Consultations where patients were only presenting for routine health examinations or contraceptive procedures or referrals were excluded (*n* = 5), leaving a total of 92 consultations. Whether patients saw their ‘usual GP’ and satisfaction scores were obtained from a post-consultation questionnaire. Problem categories were coded in a previous study.^18^

### Screening for ICEE

Matthy *et al*’s definitions of ICE^14^ were used with minor amendments (Table 1). One author screened the consultations for ‘ICEE events’, which were utterances that included any of the ICEE components. A low threshold for including anything that could be counted as an ICEE event was used in the screening process. All ICEE events were then reviewed by a second author and any disagreements discussed with an additional author until consensus was reached. The EHRs for the consultation were also screened independently for evidence of ICEE by two authors, and disagreements reviewed by a senior author.

**Table 1.** ICEE definitions

Term	Definition (adapted from Matthys *et al^14^*)
**Idea**	Are the ideas of the patient about a possible diagnosis, treatment, or prognosis expressed in the consultation? *This may include what the patient thinks might be the cause of their symptoms*.
**Concern**	Is concern (fear or worry) of the patient about a possible diagnosis, *test or* therapy present in the consultation?
**Expectation**	Is the expectation (what the patient wants) for *assessment*, treatment, a diagnosis, or a therapy present in the consultation?
**Effects on life** **(not included in Matthys** ***et al*** **)**	*Does the condition or treatment have an effect on the patient’s activities of daily living such as washing, dressing, shopping, working, sleeping, relationships, or similar*?

New additions to Matthys *et al* ^14^ are marked in italic font. An extended version with examples of this table is provided in Supplementary Table S1. ICEE = ideas, concerns, expectations, and effects on life.

### Coding

Where ≥2 ICEE components were mentioned in one sentence (for example, *'I think it’s just a cyst, but I’m concerned it might be cancer*')*,* an ICEE event for each component was recorded. One author coded the stage of the consultation that the event took place (adapted from Byrne and Long^19^), ICEE subtypes, and who initiated the event (that is, asked by GP or volunteered by patient). Effects on activities of daily living (ADL) were further classified into ‘basic’ (for example, washing and eating) and more complex ‘instrumental’ ADLs (for example, transportation and housekeeping).^20^

### Statistical analysis

Analysis was conducted in Stata (version 17.0). Each problem and consultation were given an ICEE score, calculated from the number of individual components of ICEE identified (maximum *n* = 4/4).

Multilevel mixed-effects binary and ordered logistic regression were used to assess associations (see Supplementary Tables S2–S14 for details including handling of missing data) and generate ORs with 95% CIs. For ‘in-consultation’ outcome measures (ICEE scores or components and prescriptions), clusters were problems raised by the same patient nested in patients seen by the same GP. For patient satisfaction outcomes, an additional clustering of GPs from the same practice was included, as satisfaction may have been influenced by factors outside of the consultation, such as waiting-room facilities. Multivariable modelling was used to adjust for covariables, and factors known to be associated with patient satisfaction.^21^

## Results

### Participant and consultation characteristics

A summary of participant characteristics is provided in Table 2. The mean patient and GP ages were 50 (range 18–96) and 46 years (range 32–62), respectively. There were slightly more female patients (57.6%) than male patients, and female GPs (56.5%) than male GPs. All GPs and most patients (87.0%) were from a White ethnic group.

**Table 2.** Characteristics of patients and GPs

Patients (*n* = 92)	*n*	%	GPs (*n* = 23)	*n*	%
**Patient sex**			**Doctor sex**		
Male	39	42.4	Male	10	43.5
Female	53	57.6	Female	13	56.5
**Patient age, years**			**Doctor age, years**		
18–34	28	30.4	18–34	3	13.0
35–49	14	15.2	35–49	11	47.8
50–64	28	30.4	50–64	9	39.1
≥65	20	21.7			
Not reported	2	2.2			
**IMD quintile**			**Doctor role**		
First (least deprived)	24	26.1	Partner	19	82.6
Second	19	20.7	Salaried	4	17.4
Third	13	14.1			
Fourth	13	14.1			
Fifth (most deprived)	22	23.9			
Not reported^a^	1	1.1			
**Seeing usual GP**					
Yes	27	29.3			
No	22	23.9			
Not sure	2	2.2			
I don’t have a regular GP	26	28.3			
Not reported	15	16.3			
**Post-consultation satisfaction**					
Very satisfied	63	68.5			
Satisfied	15	16.3			
Neither satisfied nor dissatisfied	0	0.0			
Dissatisfied	1	1.1			
Very dissatisfied	0	0.0			
Not reported	13	14.1			

^a^From a consultation with a practice-level median IMD category of first quintile. IMD = Index of Multiple Deprivation.

In the 92 consultations, 190 problems were raised, of which 57.9% (*n* = 110/190) were new. Two of these new problems were requests for routine health checks (for example, ‘well man’ check) and excluded from the ‘new problem’ subgroup analysis (*n* = 108 problems). The mean number of problems raised in each consultation was 2.1 (range 1–6).

### ICEE per consultation and per problem

**Figure 1.**
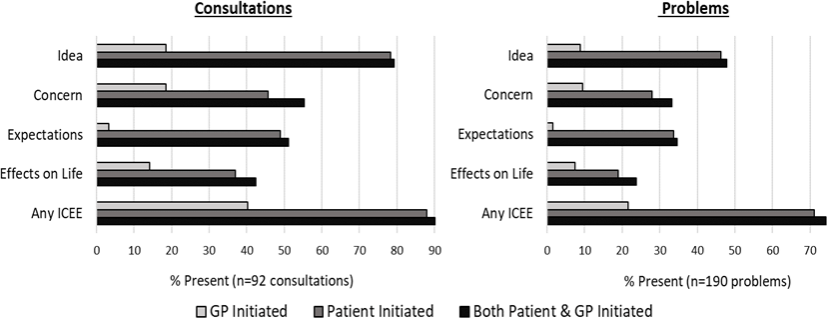
Frequency of ICEE per consultation and per problem. ICEE = ideas, concerns, expectations, and effects on life.

Figure 1 and Table 3 demonstrate the frequencies of ICEE components. Most consultations had at least one ICEE event (*n* = 83/92, 90.2%). The most frequent ICEE component per consultation was ideas (79.3%), followed by concerns (55.4%), expectations (51.1%), and effects on life (42.4%). For every ICEE component it was more common for patients to initiate the ICEE talk, most notably for expectations, raised by patients in 48.9% of consultations and by GPs in only 3.3% of consultations. Approximately three-quarters of all problems assessed had a least one ICEE component (74.2%), although this was slightly higher for new problems (82.4%).

**Table 3.** Frequency of ICEE per consultation and per problem

Category	Both patient and GP initiated		GP initiated		Patientinitiated^a^		Documented in EHR	
**Consultations (*n* = 92 total*;*** ***n* = 82 with EHR**)	***n***	**%**	***n***	**%**	***n***	**%**	***n***	**%**
Idea	73	79.3	17	18.5	72	78.3	15	18.3
Concern	51	55.4	17	18.5	42	45.7	14	17.1
Expectation	47	51.1	3	3.3	45	48.9	13	15.9
Effects on life	39	42.4	13	14.1	34	37.0	20	24.4
Any	83	90.2	37	40.2	81	88.0	44	53.7
**All problems (*n* = 190 total*;*** ***n* = 170 with EHR**)								
Idea	91	47.9	17	8.9	88	46.3	17	10.0
Concern	63	33.2	18	9.5	53	27.9	15	8.8
Expectation	66	34.7	3	1.6	64	33.7	15	8.8
Effects on life	45	23.7	14	7.4	36	18.9	21	12.4
Any	141	74.2	41	21.6	135	71.1	56	32.9
**New problems (*n* = 108 total*;*** ***n* = 97 with EHR)^b^**								
Idea	65	60.2	11	10.2	64	59.3	11	11.3
Concern	44	40.7	12	11.1	36	33.3	11	11.3
Expectation	36	33.3	2	1.9	34	31.5	4	4.1
Effects on life	25	23.1	7	6.5	20	18.5	12	12.4
Any	89	82.4	24	22.2	86	79.6	32	33.0

Whether problems were new (‘first presentations’) or not were coded in a previous study.^18^ ^a^Includes ICEE events raised by a third party accompanying the patient to the consultation. ^b^Excludes two ‘new’ problems that were requests for routine health checks. EHR = electronic health records. ICEE = ideas, concerns, expectations, and effects on life.

### ICEE events

A total of 487 utterances were put forward for a second reviewer opinion, with 400 events included in the final analysis (Table 4). Ideas (*n* = 151, 37.8%) were the most common, followed by concerns (*n* = 97, 24.3%), expectations (*n* = 86, 21.5%), and effects on life (*n* = 66, 16.5%).

**Table 4.** Subcategories of ICEE events

ICEE type	Subcategory	*n*	%	Example verbatim
**Ideas**	**Cause of symptom(s**)	85	56.3	*'I’ve just felt so tired, and I just wonder if I’m depleted in anything?'*
	**Specific diagnosis**	50	33.1	*'I think I may have torn a muscle in my shoulder.'*
	**Treatment or medication**	14	9.3	*'I don’t know if that is down to the medication, like where I’m not so aggressive towards people.'*
	**Questioned but none**	2	1.3	GP: *'Have you got any idea what it is yourself?*' Patient: *'No, I haven't.'*
**Concerns**	**Symptom(s**)	35	36.1	*'I looked at my tongue and it scared me stiff. I got black dots; I don’t know if that’s normal or what.'*
	**Severity of condition**	23	23.7	GP: *'Yeah. But you want to make sure it’s not anything else untoward going on?'*Patient: *'Yeah, or that it’s not a serious issue.'*
	**Medication related**	16	16.5	*'I don't want to have steroids and put on weight again. I'm worrying about that.'*
	**Future health**	11	11.3	*‘If I did get rid of the baby, could it affect me conceiving in the future?'*
	**Other: ADLs (*n* = 3) test results (*n* = 2**)	5	5.2	*'There was one more as well that I was worried about. When I saw the immunologist, over the summer, they said that my T cells were up.'*
	**Questioned but none**	7	7.2	GP: *'You are not worried that it is anything else going on?'* Patient: *'No.'*
**Expectations**	**Medication related**	26	30.2	*'My friend takes them* [Migraleve], *and she swears by them. I thought, I’ll ask the doctor, she knows, and we can go from there.'*
	**Investigation(s**)	19	22.1	*'She’s on about, "Ask whether you could have a check for a PSA* [prostate-specific antigen]*" whatever that is.'*
	**For other treatment**	19	22.1	*'All I want is to get it done. I'd put up with the operation.'*
	**Physical exam**	11	12.8	*'I just wondered if it would be worth just having a quick listen to my chest.'*
	**Diagnosis**	4	4.7	*'I would like to know what’s happening with my arm.'*
	**Other: referral (*n* = 3), fit note (*n* = 2), and general advice (*n* = 2**)	7	8.1	*'But I would appreciate now if you could get me in to see the gynaecologist.'*
**Effects on life**	**Work related**	18	27.3	*'I’m a taxi driver … I can drive, but it’s painful.'*
	**Basic ADL**	16	24.2	*'I find it more difficult to cut my food, to slice the chicken last night.'*
	**Instrumental ADL**	12	18.2	*'I can’t go to the gym, which is annoying. I can’t even– I used to cycle a lot and I can’t even do that.'*
	**Sleep**	10	15.2	*'I had to sleep downstairs, upright, in the night; I never had any choice in it.'*
	**Mood**	6	9.1	*'With my thrush and everything else, it’s bringing me down.'*
	**Questioned but none**	4	6.1	GP: *'Does the pain stop you doing things?'*Patient*:'I’m doing everything. I’m still carrying on. I just try to ignore it.'*

ADL = activities of daily living. ICEE = ideas, concerns, expectations, and effects on life.

Most ICEE events were initiated by the patient (*n* = 338, 84.5%) (for example, *'I think it’s possibly to do with stress at work*'), rather than the GP (*n* = 56, 14.0%) (for example, *'Have you got any idea what it is yourself?*'). Occasionally events were raised by a third party present in the room (*n* = 6, 1.5%) (for example, *'She’s stopped doing her activities because she’s constantly going to the loo*'), although more frequently patients themselves referred to a third party (*n* = 21) (for example, *'He said it was probably just like lactic acid or something'*) (data not shown).

Most ICEE events were observed in the ‘gathering information’ stage of the consultation (54.0%, Table 5). However, patient expectations — which were almost entirely initiated by the patient or a third party (*n* = 83/86) — were mostly raised in the ‘treatment planning' stage (37.2%).

**Table 5.** ICEE events by consultation stage

ICEE type	Consultation stage										
	Establishing reason		Gathering information		Delivering diagnoses		Treatment planning		Closing		Total
	*n*	Row %	*n*	Row %	*n*	Row %	*n*	Row %	*n*	Row %	*n*
**Idea**	42	27.8	87	57.6	5	3.3	15	9.9	2	1.3	151
**Concern**	17	17.5	53	54.6	6	6.2	19	19.6	2	2.1	97
**Expectation**	22	25.6	28	32.6	2	2.3	32	37.2	2	2.3	86
**Effects on life**	10	15.2	48	72.7	1	1.5	7	10.6	0	0.0	66
**Total**	91	22.8	216	54.0	14	3.5	73	18.3	6	1.5	400

ICEE = ideas, concerns, expectations, and effects on life.

### Problem and consultation variables and ICEE scores

The number of problems raised in a consultation was associated with an increased overall consultation ICEE score (OR 2.09 per increase in problems raised, 95% CI = 1.33 to 3.29, *P* = 0.001; Supplementary Table S2). Similar results were found when only patient-initiated consultation ICEE scores were included (OR 2.13, 95% CI = 1.32 to 3.44, *P* = 0.002; Supplementary Table S3) but not for GP-initiated consultation ICEE scores (*P* = 0.33; Supplementary Table S4).

The order a problem was assessed by the GP was associated with ICEE scores, with problems assessed later in the consultation having lower overall problem ICEE scores (OR 0.60 per unit increase, 95% CI = 0.41 to 0.87, *P* = 0.007; Supplementary Table S5a). This finding was repeated for both patient-initiated problem ICEE scores (OR 0.62 per unit increase, 95% CI = 0.42 to 0.90, *P* = 0.011; Supplementary Table S6) and GP-initiated problem ICEE scores (OR 0.44 per unit increase, 95% CI = 0.23 to 0.84, *P* = 0.013; Supplementary Table S7), and when only problems from consultations containing multiple problems were analysed (OR 0.59, 95% CI = 0.40 to 0.88, *P* = 0.009; Supplementary Table S5b)

Whether problems were new was not associated with ICEE scores, but acute problems were associated with higher overall problem ICEE scores than chronic problems (OR 2.98, 95% CI = 1.36 to 6.53, *P* = 0.007; Supplementary Table S5a). This finding was reproduced in patient-initiated problem ICEE scores (OR 2.62, 95% CI = 1.19 to 5.77, *P* = 0.017; Supplementary Table S6) but not GP-initiated problem ICEE scores (*P* = 0.54; Supplementary Table S7).

### GP variables and ICEE scores

Problems assessed by GPs aged ≥50 years had higher overall problem ICEE scores (OR 2.10, 95% CI = 1.07 to 4.13, *P* = 0.030; Supplementary Table S5a). Evidence for this association was similar for GP-initiated problem ICEE scores (OR 2.96, 95% CI = 1.11 to 7.93, *P* = 0.030; Supplementary Table S7), but weaker for patient-initiated problem ICEE scores (OR 1.90, 95% CI = 0.97 to 3.71, *P* = 0.060; Supplementary Table S6). Whether patients reported they were seeing their usual GP was not associated with any ICEE scores (see Supplementary Tables S2–S7).

### Patient variables and ICEE scores

Patient sex and ethnic group were not associated with ICEE scores (see Supplementary Tables S2–S7). Problems raised in consultation with older patients (aged ≥75 years) were associated with lower overall problem ICEE scores (OR 0.40, 95% CI = 0.16 to 0.98, *P* = 0.046; Supplementary Table S5a); evidence for this association was much weaker for patient-initiated ICEE scores (OR 0.50, 95% CI = 0.20 to 1.24, *P* = 0.13; Supplementary Table S6), and no association was seen with GP-initiated ICEE sores (*P* = 0.93; Supplementary Table S7). This pattern was replicated when comparing the most socioeconomically deprived group with the least for overall problem ICEE scores (OR 0.39, 95% CI = 0.17 to 0.92, *P* = 0.032; Supplementary Table S5a), with only very weak evidence for patient-initiated problem ICEE scores (OR 0.49, 95% CI = 0.21 to 1.15, *P* = 0.10; Supplementary Table S6) and no evidence observed for GP-initiated problem ICEE scores (*P* = 0.62; Supplementary Table S7).

### ICEE documentation and prescriptions

Despite being the least frequently raised component of ICEE, effects on life were the most frequently documented in the patient medical records (see Table 3). There were no significant associations between the presence of any ICEE components and prescription frequencies for new problems (see Supplementary Table S8).

### ICEE and patient satisfaction

There were no associations between post-consultation patient satisfaction and any consultation ICEE scores (see Supplementary Tables S9–S11). In multivariable modelling with each of the separate ICEE components, patient ideas were associated with increased satisfaction (OR 10.74, 95% CI = 1.60 to 72.0, *P* = 0.014; Supplementary Table S12), whereas the opposite was true for concerns (OR 0.14, 95% CI = 0.02 to 0.86, *P*=0.034; Supplementary Table S12). The association of higher satisfaction remained when only patient-initiated ideas were assessed (OR 6.36, 95% CI = 1.16 to 34.86, *P* = 0.033; Supplementary Table S13) but not for GP-initiated ideas (*P* = 0.20; Supplementary Table S14). There was no association when only patient-initiated or GP-initiated concerns were analysed (*P* = 0.10 and *P* = 0.58; Supplementary Tables S13–S14).

## Discussion

### Summary

In most consultations, patients raised at least one ICEE component, most commonly ideas about the cause of their symptoms. Most ICEE events occurred in the 'gathering information’ stage of the consultation. GPs initiated ICEE dialogue in less than half of the consultations, asking mostly about patient ideas and concerns but rarely about expectations. The larger the number of problems discussed in a consultation, the more likely it was for more ICEE components to be raised. Problems that were acute, assessed by older GPs, or assessed earlier in the consultation were associated with higher ICEE scores. There was weak evidence that problems raised by older patients or patients from more deprived backgrounds had lower ICEE scores. Mentions of patients’ ideas and patients’ concerns were associated, respectively, with increased and decreased post-consultation satisfaction.

### Strengths and limitations

The screening for ICEE events was robust, with a low threshold for a second review of anything that could be an ICEE event. Using recordings and transcripts is advantageous over direct field observation^14^ as it allows multiple reviews of consultation content by multiple coders.

While the study demonstrated associations between patient satisfaction and the presence of ideas and concerns, these results should be interpreted with caution. First, all apart from one patient were satisfied or very satisfied with their consultation and one can question the relevance of comparing these two ratings. However, an interview study of patients reporting these two post-consultation scores has shown a meaningful difference akin to 'acceptable' (satisfied) and 'outstanding' (very satisfied).^22^ Given previous research^23^ has reported similar skewed post-GP-consultation satisfaction distributions (77% ‘very satisfied’), it was felt the dichotomous analysis of ‘very satisfied’ or not was appropriate for this distribution. Second, neither association was detected when only GP-initiated ICEE components were assessed, so these associations may have been related to how ICEE were subsequently dealt with by the clinician. Third, the models were limited by the sample size available, and the development of multiple exploratory models increases the probability of producing a spuriously significant result. Most attention should be paid to the overall ICEE models rather than patient- or GP-initiated specific results that could be impacted by one another (for example, if a patient has already vocalised their ideas there would be no need for GPs to directly ask this question). Finally, patient satisfaction is known to be affected by factors both within and outside the consultation such as the helpfulness of reception staff.^24^ While adjustments for covariates were made, there may have been additional confounding factors, such as patients’ health literacy,^25^ and clinicians' ‘positive approach’ and display of empathy, which have been linked to increased satisfaction.^26^

The analysis was limited to consultations that contained at least one new problem from 23 GPs in the West of England and therefore is only reflective of a subset of GP–patient interactions. An association was not found between ICEE scores and if patients were consulting their usual GP. However, analysis of these data were limited by almost one-third of patients reporting either not having a regular GP or not knowing who their regular GP was. A larger sample size is required to draw more firm conclusions on the relationship between ICEE and other patient or clinician factors.

### Comparison with existing literature

Observational studies of ICEE have been reported outside of the UK. Matthys *et al* ^14^ reported on ICE in 613 GP–patient consultations in 36 Belgian practices observed in 2005. They found that in only 20% of consultations were all three ICE components expressed, compared with 34% in the current study. Unlike Matthys *et al*, the present study did not find an association between the presence of concerns or expectations and fewer prescriptions.

In 2006, Towle *et al* ^13^ examined audio-recorded consultations involving 198 patients and six family clinicians in Canada. Most ICE events occurred in the ‘problem identification’ rather than ‘problem management’ phase, consistent with the present study. Although Towle found clinicians were competent at eliciting ICE, ‘rarely’ were all three components explicitly addressed. Clinicians reported they made a conscious decision whether to ask these questions citing a lack of time, concerns about repetition, discomfort dealing with patients’ emotional state, and that it could be equivalent to opening ‘Pandora’s box’.

Freilich *et al* ^27^ used questionnaires in 2015 to compare perceptions of whether ICE emerged during consultations with GPs, district nurses, and physiotherapists in Sweden. Patient and GP assessments of ICE components were roughly concordant: ideas (76% patient versus 73% GP), concerns (23% versus 17%), expectations (31% versus 40%). These percentages are lower than the present study's findings for concerns (55%) and expectations (51%), but similar for ideas (79%).

### Implications for research and practice

Future studies could use patient post-consultation questionnaires to explicitly ask whether patients thought they had discussed their ICEE and whether these were adequately assessed. Likewise, a clinician post-consultation questionnaire could be used to evaluate researchers’ and clinicians’ concordance with respect to classifying ICEE.

Post-COVID-19 pandemic, alternatives to face-to-face consultation in primary care have become more common.^28^ This study provides a benchmark of ICEE frequencies in face-to-face consultations that could be compared with other consultation modalities.

Further research on how ICEE components are initiated and responded to, rather than just their presence, may provide deeper insights into their impact on patient outcomes. Further analysis to understand why patients in the study who mentioned their concerns were less satisfied would be helpful when training new GPs. One possible explanation is that patients voicing concerns were more anxious pre-consultation, and less easy to reassure. Another explanation could be dissatisfaction with how their concerns were subsequently addressed. The use of concise pre-consultation forms has shown promising initial results in identifying patients' concerns, enabling clinicians to proactively address them and improve satisfaction.^29^

The finding that problems assessed later in the consultation had fewer ICEE components raised aligns with previous research demonstrating additional problems had less safety-netting advice^30^ and only increased consultation duration by 2 minutes per extra problem,^31^ adding to the evidence multi-problem consultations may be higher risk or less satisfactory for patients and GPs.

Finally, the finding that older GPs discussed more ICEE components per problem than their younger counterparts, contrasts with a previous study that showed that younger GPs provided more ‘safety-netting advice’.^18^ This warrants further investigation into how routine practice is changing with time and any impacts this will have on the quality and safety of consultations.

## Data

The 'One in a million: a study of primary care consultations' (https://doi.org/10.5523/bris.l3sq4s0w66ln1x20sye7s47wv) is a controlled dataset due to participants potentially being identifiable. To access the data please see https://www.bristol.ac.uk/primaryhealthcare/researchthemes/one-in-a-million/accessing-the-data for more information.
